# Microbial Community Analysis of Anaerobic Enrichment Cultures Supplemented with Bacterial Peptidoglycan as the Sole Substrate

**DOI:** 10.1264/jsme2.ME20002

**Published:** 2020-09-12

**Authors:** Samia Quaiyum, Kensuke Igarashi, Takashi Narihiro, Souichiro Kato

**Affiliations:** 1 Division of Applied Bioscience, Graduate School of Agriculture, Hokkaido University, Kita-9 Nishi-9, Kita-ku, Sapporo 060–8589, Japan; 2 Bioproduction Research Institute, National Institute of Advanced Industrial Science and Technology, 2–17–2–1 Tsukisamu-Higashi, Toyohira-ku, Sapporo 062–8517, Japan; 3 Bioproduction Research Institute, National Institute of Advanced Industrial Science and Technology, 1–1–1 Higashi, Tsukuba 305–8567, Japan

**Keywords:** peptidoglycan, microbial necromass, anaerobic degradation, methanogenesis, community analysis

## Abstract

Methanogenic microbial communities were enriched from rice paddy soil and anaerobic digester sludge using peptidoglycan purified from gram-negative *Escherichia coli* or gram-positive *Micrococcus luteus* as the sole substrate. Methane production data suggested the anaerobic degradation of peptidoglycan and also that peptidoglycan from *E. coli* had lower degradability. The community structures of enrichment cultures fed peptidoglycan from *E. coli* or *M. luteus* were similar, but distinctly different. A number of phylogenetically novel and uncultured bacteria, particularly in the phyla Bacteroidetes, WWE1, Armatimonadetes, and Verrucomicrobia, dominated the enrichment cultures, suggesting their involvement in anaerobic peptidoglycan degradation.

Organic compounds originally generated from carbon dioxide mainly by photosynthetic organisms are eventually broken down to carbon dioxide by both aerobic and anaerobic microorganisms; this process constitutes the global carbon cycle. Although microorganisms are generally regarded as final consumers/degraders, the production and degradation of organic matter during their growth and after their death, respectively, also significantly contribute to carbon cycles in natural environments (Ogawa *et al.*, 2001; Dong *et al.*, 2018; Liang *et al.*, 2019). The degradation of microbial cells is also important in some engineering environments (Appels *et al.*, 2008; Hanajima *et al.*, 2015; Chatzigiannidou *et al.*, 2018). For example, the degradation of waste activated sludge, of which bacterial cells are a major component, by anaerobic microorganisms (*i.e.*, anaerobic digestion) is a well-established strategy to reduce solid waste derived from municipal wastewater treatment in an environmentally friendly manner (Appels *et al.*, 2008).

Bacterial cells are composed of complex organic compounds, including proteins, nucleic acids, polysaccharides, and lipids. Among cell components, peptidoglycan, the main constituent of the cell wall, is recalcitrant to microbial degradation, which is regarded as the rate-limiting step in the turnover of organic matter derived from bacterial cells (Jørgensen *et al.*, 2003; Nagata *et al.*, 2003; Scheffers and Pinho, 2005). Peptidoglycan is a polymer consisting of polysaccharides and peptides that forms a mesh-like layer outside the cytoplasmic membrane, which confers structural strength to bacterial cells. Polysaccharide chains consist of alternating residues of β-(1,4)-linked N-acetylglucosamine and N-acetylmuramic acid and are cross-linked by short peptides composed of four or five amino acids. The composition of short peptides varies among bacterial species, particularly between gram-positive and gram-negative bacteria (Vollmer *et al.*, 2008a). Although fine structures (*e.g.*, the length of glycan chains and type and degree of glycan-peptide and peptide-peptide cross-linking) change depending on growth properties, the major structures specific for bacterial types (compositions of amino acids) do not change regardless of growth conditions (Vollmer *et al.*, 2008a).

Previous studies investigated the fate of peptidoglycan in aerobic environments, and demonstrated that peptidoglycan was eventually degraded and its turnover rate was markedly lower than those of other cellular components (Jørgensen *et al.*, 2003; Nagata *et al.*, 2003). Most bacteria have peptidoglycan-hydrolyzing enzymes because peptidoglycan needs to be partially degraded and remodeled during the elongation and division of cells (Vollmer *et al.*, 2008b; Vermassen *et al.*, 2019). However, these enzymes generally function intracellularly and do not contribute to the degradation of extracellular peptidoglycan. Some aerobic bacteria have been reported to excrete peptidoglycan-hydrolyzing enzymes extracellularly and/or utilize extracellular peptidoglycan as their growth substrate (Vollmer *et al.*, 2008b; Jørgensen *et al.*, 2010; Vermassen *et al.*, 2019). Moreover, there is currently no information on the fate of peptidoglycan, peptidoglycan-degrading microorganisms, or extracellular peptidoglycan-hydrolyzing enzymes in anaerobic environments, while the degradation of bacterial cells has been shown to markedly contribute to the carbon cycle, even in anaerobic environments (Dong *et al.*, 2018; Liang *et al.*, 2019).

In the present study, microbial communities were enriched under anaerobic, methane (CH_4_)-producing conditions with peptidoglycan as the sole substrate using rice paddy soil and sludge from an anaerobic digester as the microbial sources in order to investigate the peptidoglycan degradation capability of anaerobic microorganisms. Furthermore, the microbial community structures of the enrichments were examined using a next-generation sequencing analysis of 16S rRNA gene amplicons to identify the types of microorganisms contributing to anaerobic peptidoglycan degradation.

Peptidoglycan-degrading microbial communities were enriched from natural (rice paddy, hereafter “RP enrichments”) and engineered (an anaerobic digester, hereafter “AD enrichments”) anaerobic environments. Enrichment cultures were performed under methanogenic conditions because CH_4_ is the main product of anaerobic metabolism in these environments. Rice paddy soil was collected from an experimental farm at the Hokkaido Agricultural Research Center of the National Agriculture and Food Research Organization (Sapporo, Hokkaido, Japan). Sludge was collected from an anaerobic digester treating waste activated sludge operated at 42°C in Japan (Mei *et al.*, 2017). Two milliliters of slurry of either rice paddy soil or sludge was inoculated into vials (68-mL capacity) containing 18‍ ‍mL of freshwater medium with the following ingredients (L^–1^): 0.54‍ ‍g NH_4_Cl, 0.13‍ ‍g KH_2_PO_4_, 0.24‍ ‍g MgCl_2_·6H_2_O, 0.15‍ ‍g CaCl_2_·2H_2_O, 2.52‍ ‍g NaHCO_3_, 0.1‍ ‍g Bacto yeast extract, 0.1‍ ‍g Bacto proteose peptone, and 1‍ ‍mL each of a trace element solution and vitamin solution (Sekiguchi *et al.*, 2000). The medium was supplemented with the powder of activated carbon (diameters of 37 to 149‍ ‍μm, Sigma-Aldrich) at a final concentration of 1‍ ‍g L^–1^ to promote the downstream reactions of methanogenesis (mainly syntrophic degradation of organic acids) (Kato *et al.*, 2012). Peptidoglycan purified from gram-negative *Escherichia coli* (hereafter “ECPG”) or gram-positive *Micrococcus luteus* (hereafter “MLPG”) were prepared by a method described elsewhere (Ziamko and Okulich, 2014). Briefly, *E. coli* and *M. luteus* cells were treated with 30% aqueous phenol at 65°C for 20‍ ‍min to remove lipids, (lipo)polysaccharides, (lipo)proteins, and other components associated with peptidoglycan. The pellet obtained by centrifugation was further treated with 3% acetic acid at 100°C for 3 h to eliminate trace amounts of lipopolysaccharides. The precipitates obtained by centrifugation were washed three times by sterile distilled water and then subjected to dialysis against 0.05 M sodium acetate solution (pH 5.8) for 3 days at 22°C to remove low molecular weight contaminants. The precipitates obtained by centrifugation were dried at 50°C to obtain peptidoglycan powder. It is important to note that this procedure is based on the protocol for obtaining high-purity peptidoglycan required for the chemical classification of bacteria, while trace contamination (particularly teichoic acids from Actinobacteria) is inevitable (Ziamko and Okulich, 2014). ECPG or MLPG in an insoluble powder state was added to the freshwater medium at a final concentration of 50‍ ‍g dry weight L^–1^ as the sole substrate. Cultures were incubated at 30°C (for RP enrichments) or 42°C (for AD enrichments) under a N_2_:CO_2_ atmosphere (80:20‍ ‍[v/v]) without shaking. CH_4_ production was assessed using gas chromatography, as described previously (Kato *et al.*, 2014). After an incubation for approximately 2 months, 2‍ ‍mL of each enrichment was subcultured in 18‍ ‍mL of fresh medium. All culture experiments were conducted in triplicate and statistically analyzed using the Student’s *t*-test.

After three successive subcultures to eliminate the influence of organic compounds derived from the inocula, CH_4_ production from peptidoglycan was measured (Fig. 1). The RP and AD enrichments produced CH_4_ at similar amounts and rates; they produced 52–56 and 17–23‍ ‍mmol L^–1^ of CH_4_ from MLPG and ECPG, respectively, within 2 months. These CH_4_ production rates were significantly higher than those in the no substrate controls (4.5–5.5‍ ‍mmol L^–1^) (*P*<0.01), in which CH_4_ was produced only from organic matter carried over from the inoculated samples (soil and sludge) and derived from trace amounts of yeast extract and peptone. These results suggest that at least some peptidoglycan was degraded and converted into CH_4_ under anaerobic conditions. In the present study, we were unable to clarify the extent to which peptidoglycan was degraded. This was mainly attributed to the difficulties associated with tracking the fate of peptidoglycan because it is a complex polymer composed of multiple types of sugars and amino acids, peptidoglycan used in the present study was an insoluble powder, and microorganisms growing on degraded peptidoglycan newly generate their own peptidoglycan. The carbon balance was estimated by approximating the chemical composition of peptidoglycan to be [CH_2_O] and assuming that peptidoglycan degradation proceeded according to the equation 2[CH_2_O] → CH_4_+CO_2_. Based on this estimation, we roughly estimated that up to 10% of peptidoglycan was converted into CH_4_ and CO_2_ during the 2-month incubation. Although the degradation rate is not so high (likely due to the recalcitrance of peptideglycan), this estimation supports the occurrence of anaerobic peptidoglycan degradation. (Jørgensen *et al.*, 2003; Nagata *et al.*, 2003). Further investigations, such as enrichment cultures using ^13^C-labeled peptidoglycan, followed by the detection of labeled CH_4_ and CO_2_, are required to provide direct evidence of microbial peptidoglycan degradation and elucidate behaviors, such as the carbon balance, in more detail.

RP and AD enrichments both produced more CH_4_ from MLPG than from ECPG, which suggests that ECPG (and potentially peptidoglycan from gram-negative bacteria) is more recalcitrant to microbial degradation. This is consistent with previous findings obtained in aerobic environments; for example, peptidoglycan derived from gram-negative bacteria was found to be more resistant to attack by aerobic bacteria (Jørgensen *et al.*, 2003). One factor potentially affecting degradability is structural differences between MLPG and ECPG. The most prominent structural difference is the constituents of the short peptide: 2,6-diaminopimelic acid is present in ECPG (and most gram-negative bacteria), but not in MLPG (and most gram-positive bacteria) (Vollmer *et al.*, 2008a), which may result in different degradabilities. Furthermore, trace impurities in the substrate peptidoglycan (*e.g.*, teichoic acids in MLPG) may affect methane production. Further studies, including assessments of the degradability of peptidoglycan from more diverse bacteria and an analysis of intermediate products from the degradation of peptidoglycan, will provide insights into the mechanisms underlying differences in the degradability of peptidoglycan from gram-negative and gram-positive bacteria.

To identify the microorganisms that dominated the enrichment cultures supplemented with peptidoglycan, a microbial community analysis was conducted as described previously (Tu *et al.*, 2019). Since the contribution of eukaryotes (protozoa and fungi) was expected to be negligible under strictly anaerobic conditions and eukaryotic cells were not detected by microscopic observations, only bacteria and archaea were targeted for the community analysis. DNA was extracted using the FAST DNA Spin Kit for Soil (MP Biomedicals) and the 16S rRNA gene V4 region was amplified by PCR using Phusion Hot Start II High-Fidelity DNA Polymerase (Thermo Fisher Scientific) with the primer pair 515′F/805R (Hugerth *et al.*, 2014) with adaptor and index sequence tags under the following thermal conditions: initial thermal denaturation at 98°C for 30‍ ‍s, followed by 25 cycles of heat denaturation at 98°C for 10‍ ‍s, annealing at 55°C for 20‍ ‍s, and extension at 72°C for 30 s. PCR products were subjected to agarose gel electrophoresis to confirm fragment lengths and then purified with the QIAquick PCR Purification Kit (Qiagen). Purified PCR products were subjected to a sequencing analysis with an Illumina MiSeq platform (Illumina) by Hokkaido System Science to generate Illumina shotgun paired-end (2×301 bp) sequence libraries. Raw 16S rRNA sequence data were adaptor trimmed at the 3′ end to remove adaptor sequences (cutadapt 1.1), quality trimmed (Trimmomatic v. 0.32; TRAILING:20 MINLEN:50), and individual read pairs were overlapped to form single synthetic reads (fastq-join v. 1.1.2-537; 8% maximum difference, 6‍ ‍minimum overlaps; https://github.com/brwnj/fastq-join). The reads obtained were clustered with the UCLUST algorithm using a ≥97% sequence identity cut-off with MacQIIME 1.9.1. Representative sequences of each operational taxonomic unit (OTU) were aligned using PyNAST and chimeric sequences were removed using ChimeraSlayer. A total of 58,274 16S rRNA gene reads (3,052–5,334 per sample) were retrieved and classified into 4,711 OTUs. Phylogenetic distribution patterns based on the relative abundance of major OTUs (>1% in at least one condition) and a principal component analysis based on these data showed that the community structures of the triplicate enrichment cultures were very similar, while those of different enrichment cultures and of the inoculants markedly differed from each other (Fig. S1). Therefore, the average values of the community analysis data of triplicate cultures were used in further analyses.

Phylogenetic distribution patterns at the phylum level showed that microbial community structures markedly changed during enrichment cultures (Fig. S2). The phyla that increased during the enrichment cultures were Euryarchaeota, Bacteroidetes, Firmicutes, Thermotogae (only in +ECPG enrichments), Chlorobi (only in RP enrichments), Armatimonadetes (only in +MLPG enrichments), and Verrucomicrobia (only in the RP+ECPG enrichment).

A hierarchical cluster analysis was performed on the relative abundance patterns of major OTUs (>1% in at least‍ ‍one condition) using Morpheus software (https://software.broadinstitute.org/morpheus) to infer the microbial species involved in anaerobic peptidoglycan degradation (Fig. 2). The major OTUs were classified into clusters I to VI. Cluster I contained OTUs dominant only in the original microflora of rice paddy soil and presumably unrelated to peptidoglycan degradation. The OTUs in cluster II dominated the original microflora of the anaerobic digester sludge. It is important to note that cluster II may include anaerobic peptidoglycan degraders because the anaerobic digester used in the present study treated waste activated sludge containing bacterial cells as one of the major components. Some OTUs in cluster II also dominated the enrichment cultures (*e.g.*, OTU2653, discussed below). Clusters III, IV, V, and VI contained OTUs dominant in the AD+MLPG, RP+MLPG, RP+ECPG, and AD+ECPG enrichments, respectively, and are expected to contain anaerobic peptidoglycan degraders. However, these clusters may also contain OTUs involved in downstream reactions, such as the fermentation of monosaccharides and amino acids, the oxidation of fermentation products, such as organic acids, and methanogenesis. OTUs closely related to a syntrophic organic acid–oxidizing bacterium (OTU1328, related to *Syntrophomonas zehnderi* with 96% identity) and an aceticlastic methanogen (OTU1874, related to *Methanosarcina mazei* with 100% identity) were classified into clusters III and VI, respectively. Microorganisms involved in these downstream reactions are expected to dominate the enrichments irrespective of the peptidoglycan sources. However, microbial community structures clearly differed between the +ECPG and +MLPG enrichments. Furthermore, some OTUs specifically dominated either enrichment, which suggests the presence of microorganisms specialized for the degradation of ECPG or MLPG. Furthermore, some OTUs dominated both the RP and AD enrichments despite differences in the microorganisms contained in the inoculants and their incubation temperatures (30 and 42°C, respectively). OTU37 (related to *Clostridium hydrogeniformans* with 96% identity) and OTU4629 (related to *Geotoga aestuarianus* with 83% identity) in cluster VI specifically dominated the RP+ECPG and AD+ECPG enrichments, which suggests that these bacteria specialize in the degradation of peptidoglycan from gram-negative bacteria.

A number of OTUs dominant in enrichment cultures were assigned as phylogenetically novel bacteria, that is, they showed low sequence identities to known bacterial species (OTUs with <90% identities to isolated species are highlighted in bold in Fig. 2). Seven out of 14 Bacteroidetes OTUs dominantly detected in the enrichment cultures had identities of <90% to known Bacteroidetes species. Members of Bacteroidetes are frequently detected from various anaerobic environments, such as anaerobic digesters, rice paddies, and animal intestines, and are considered to mainly contribute to the fermentation of biomass polymers (Rivière *et al.*, 2009; White *et al.*, 2014; Mei *et al.*, 2017; Hassa *et al.*, 2018). The present results suggest that phylogenetically novel Bacteroidetes play a pivotal role in the degradation of bacterial cell walls.

Furthermore, some dominant OTUs in the enrichment cultures were classified into phyla with no or only a few isolated strains, namely, candidate or rare phyla, respectively (highlighted in red letters in Fig. 2). WWE1 is a candidate phylum with no isolated strain. Although WWE1 bacteria are frequently found in various anaerobic environments, particularly anaerobic digesters (Chouari *et al.*, 2005; Lee *et al.*, 2017; Mei *et al.*, 2017), their function remains unknown. Two OTUs in the phylum WWE1, OTU2653 and OTU3394 (related to ‘*Candidatus* Cloacamonas acidaminovorans’ with 89 and 99% identities, respectively), were dominantly detected from all enrichment cultures supplemented with any peptidoglycan and from the AD original microflora, suggesting their involvement in peptidoglycan degradation. Armatimonadetes is a rare phylum with only a few aerobic isolates (Tamaki *et al.*, 2011). Although members of Armatimonadetes have often been detected from anaerobic environments, such as anaerobic digesters (Shin *et al.*, 2016; Zhang *et al.*, 2019), their ecophysiology in anaerobic environments remains unknown. The present results showed that two Armatimonadetes OTUs (OTU1437 related to *Oxalophagus oxalicus* with 85% identity and OTU286 related to *Ralstonia solanacearum* with 82% identity) were specifically enriched in AD+MLPG and RP+MLPG, respectively, suggesting their involvement in the degradation of peptidoglycan from gram-positive bacteria. Verrucomicrobia is also a rare phylum, in that there are not many isolated species. However, Verrucomicrobia species have been dominantly detected from anaerobic soil and digester sludge (Bergmann *et al.*, 2011; Mei *et al.*, 2017). Although most Verrucomicrobia isolates are obligate or facultative aerobes, several anaerobic species have also been isolated. For example, *Akkermansia muciniphila*, a Verrucomicrobia species isolated from human feces, fermentatively degrades mucin (glycosylated proteins produced by animals) under anaerobic conditions (Derrien *et al.*, 2004). In the present study, one Verrucomicrobia OTU (OTU1710 related to *Luteolibacter gellanilyticus* with 84% identity) was specifically enriched in the RP+ECPG culture, implying that members of this phylum degrade peptidoglycan from gram-negative bacteria.

In conclusion, the present study is the first to successfully enrich methanogenic microbial communities with peptidoglycan as the sole substrate. Peptidoglycan from gram-negative *E. coli* and that from gram-positive *M. luteus* differ in degradability and the microbial species involved in their degradation. We assume that phylogenetically diverse bacteria, particularly uncultured bacteria with high phylogenetic novelty, are involved in anaerobic peptidoglycan degradation. Further studies, such as a stable isotope probing analysis using ^13^C-labeled peptidoglycan, the identification of peptidoglycan hydrolases by metaproteomics coupled with activity staining, and the isolation of peptidoglycan degraders will contribute to the identification of peptidoglycan degraders and the elucidation of their molecular mechanisms. The isolation of anaerobic bacteria using peptidoglycan as the growth substrate may be an effective strategy for recovering uncultured anaerobes with high phylogenetic novelty. Further studies on anaerobic peptidoglycan degradation will provide us with a more detailed understanding of carbon cycles in natural environments and may lead to the development of more efficient methods for the digestion of waste activated sludge.

## Nucleotide sequence accession numbers

The sequence data obtained in the present study have been deposited in DDBJ/EMBL/GenBank under the accession numbers DRA007911 and DRA009380.

## Citation

Quaiyum, S., Igarashi, K., Narihiro, T., and Kato, S. (2020) Microbial Community Analysis of Anaerobic Enrichment Cultures Supplemented with Bacterial Peptidoglycan as the Sole Substrate. *Microbes Environ ***35**: ME20002.

https://doi.org/10.1264/jsme2.ME20002

## Supplementary Material

Supplementary Material

## Figures and Tables

**Fig. 1.**
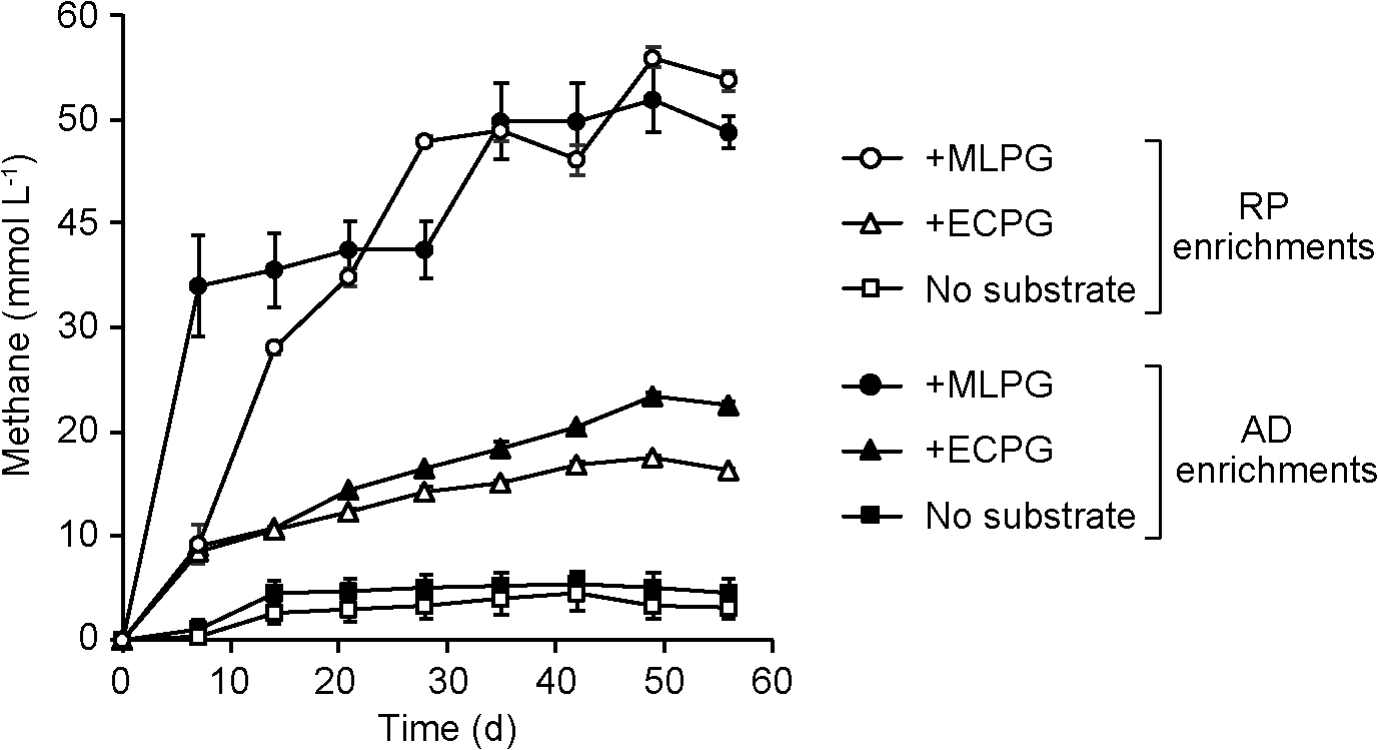
Methanogenesis by microbial communities enriched from rice paddy soil (RP) and sludge from an anaerobic digester (AD). The plot shows results from enrichment cultures after three successive subcultures supplemented with peptidoglycan purified from *Micrococcus luteus* (MLPG) and *Escherichia coli* (ECPG), as well as no substrate controls (without any subcultures). Data are presented as the means of three independent cultures. Error bars represent standard deviations.

**Fig. 2.**
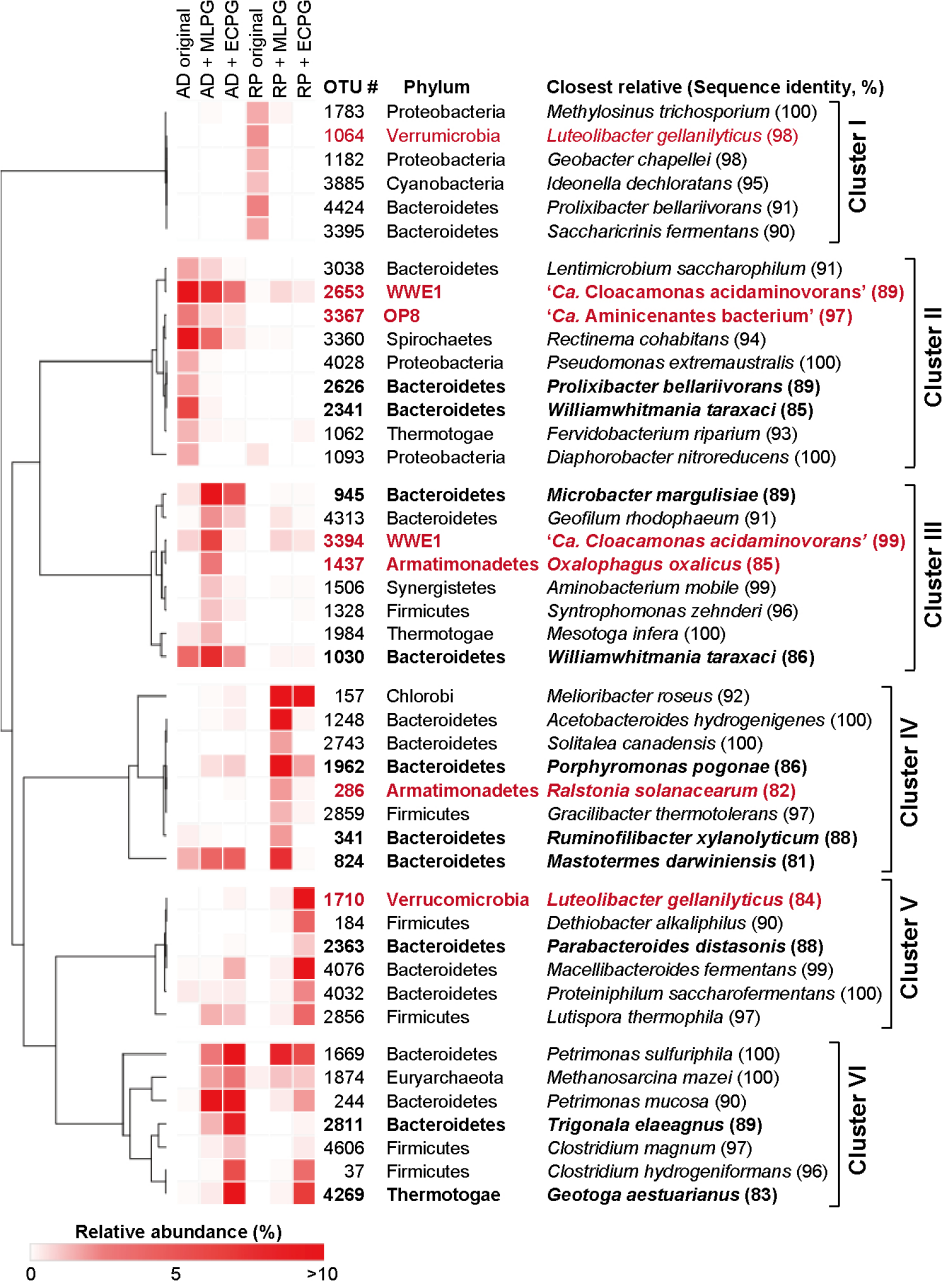
Phylogenetic distribution of enrichment cultures (and the original microflora) derived from an anaerobic digester (AD) and rice paddy soil (RP) supplemented with peptidoglycan purified from *Micrococcus luteus* (MLPG) and *Escherichia coli* (ECPG). The relative abundance patterns of the dominant OTUs (>1% in at least one condition) were subjected to a cluster analysis and are presented as a heatmap. The color intensity scale indicates the relative abundance of each OTU. The identification numbers, classified phyla, and closest relatives (sequence identity, %) of each OTU are shown. OTUs with <90% sequence identities to verified isolated species are highlighted in bold. OTUs classified into candidate or rare phyla (WWE1, OP8, Armatimonadetes, and Verrucomicrobia) are highlighted in red letters.
