# Supplementary material for: Microbial Community Analysis of Anaerobic Enrichment Cultures Supplemented with Bacterial Peptidoglycan as the Sole Substrate
Source: Microbes Environ. 2020 Sep 12;35(3):ME20002. doi: 10.1264/jsme2.ME20002 (PMC7511781; doi:10.1264/jsme2.ME20002)

Supplemental materials

Microbial community analysis of anaerobic enrichment cultures supplemented with bacterial peptidoglycan as the sole substrate

Samia Quaiyum[1,2], Kensuke Igarashi[2], Takashi Narihiro[3], Souichiro Kato[1,2,*]

[1]*Division of Applied Bioscience, Graduate School of Agriculture, Hokkaido University, Kita-9 Nishi-9, Kita-ku, Sapporo 060-8589, Japan*; [2]*Bioproduction Research Institute, National Institute of Advanced Industrial Science and Technology, 2-17-2-1 Tsukisamu-Higashi, Toyohira-ku, Sapporo 062-8517, Japan*; [3]*Bioproduction Research Institute, National Institute of Advanced Industrial Science and Technology, 1-1-1 Higashi, Tsukuba 305-8567, Japan*.

Figs S1-S2

Fig. S1. Confirmation of reproducibility of enrichment cultures performed in triplicate. (A) Phylogenetic distribution of the enrichment cultures (and the original microflora) derived from an anaerobic digester (AD) and rice paddy soil (RP) supplemented with peptidoglycan purified from *Micrococcus luteus* (MLPG) and *Escherichia coli* (ECPG) at the OTU level. Only the dominant OTUs (>1% in at least one condition) are shown. (B) The result of principal component analysis using the community analysis data shown in A.

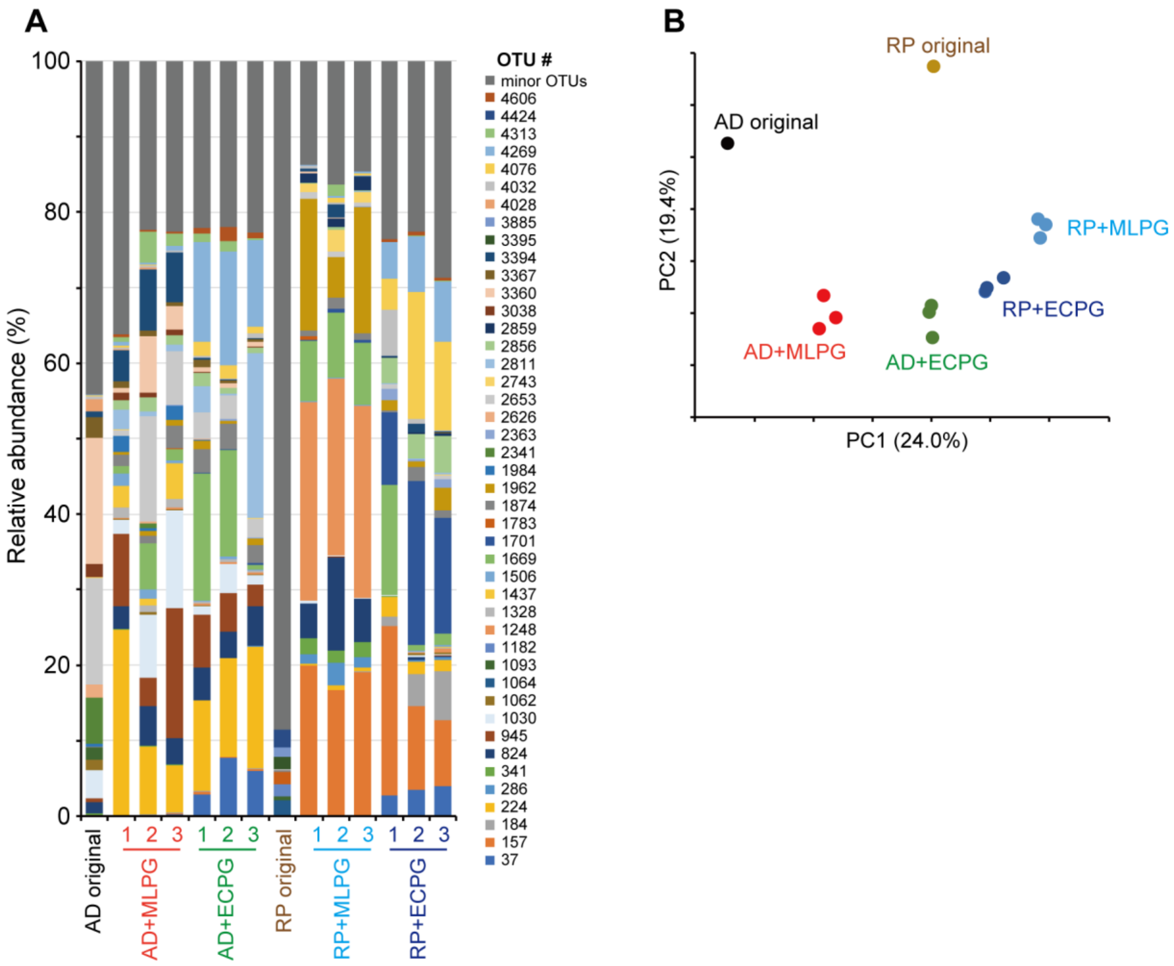

Fig. S2. Phylogenetic distribution of the enrichment cultures (and the original microflora) derived from an anaerobic digester (AD) and rice paddy soil (RP) supplemented with peptidoglycan purified from *Micrococcus luteus* (MLPG) and *Escherichia coli* (ECPG) at the phylum level. Only the dominant phyla (>3% in at least one condition) are shown. The phyla that increased in the enrichment cultures are highlighted by red borders and letters.

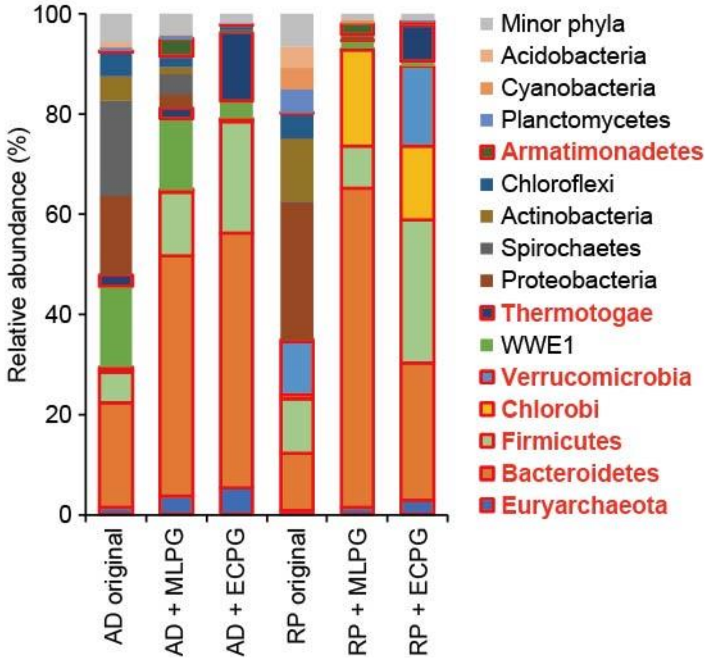

Supplement: Supplementary file 1 — Supplementary Material [file 35_20002_s1.pdf]
